# Role of primary bile salts in the regulation of sinusoidal substrate uptake in rat liver

**DOI:** 10.1186/2047-783X-19-S1-S23

**Published:** 2014-06-19

**Authors:** Stefanie Kluge, Olga Domanova, Thomas Berlage, Dieter Häussinger, Ralf Kubitz

**Affiliations:** 1 Clinic of Gastroenterology, Hepatology and Infectious Diseases, Heinrich Heine University, 40225 Düsseldorf, Germany; 2 Fraunhofer Institute for Applied Information Technology FIT, Schloss Birlinghoven, 53754 Sankt Augustin, Germany; 3 RWTH Aachen, Department of Computer Science, 52056 Aachen, Germany; 4 Bonn-Aachen International Centre for Information Technology B-IT, 53113 Bonn, Germany

## Background

Bile salts undergo enterohepatic circulation which is essential for bile salt homeostasis. From the biliary tract, they are excreted into the small intestine, absorbed into the blood and transported back to the liver. The sinusoidal uptake systems of the liver efficiently extract bile salts and other substrates from portal blood. In rat liver, these systems include Ntcp (sodium taurocholate cotransporting polypeptide) and the organic anion transporting polypeptides Oatp1a1, Oatp1a4 and Oatp1b2. While Ntcp represents the major basolateral uptake transporter for conjugated bile salts, the Oatps have broad substrate spectra including estrogen- or leukotriene-conjugates. Their transport function is regulated by long-term and short-term mechanisms. Long-term adaptation of bile salt transporters involve changes at the level of gene expression and transporter degradation, while short-term regulation includes covalent transporter modifications, substrate availability and rapid endo- and exocytosis of transporter-containing vesicles (reviewed in [1]). Ntcp as well as Oatp1a1 and Oatp1a4 underlie short-term control which has been demonstrated in several *in vitro* models. Hypoosmolarity or cAMP for instance increase the Ntcp-dependent bile salt uptake (increase of V_max_) within minutes by translocation of intracellular Ntcp to the plasma membrane [2,3]. In contrast, rapid clathrin-dependent endocytosis of Ntcp was demonstrated only recently by activation of PKC with phorbolesters [4,5].

However, to date *in vivo*-studies on short-term regulation of basolateral transporters are rare. Bile salt inflow to the liver may increase considerably under pathophysiological conditions or postprandial under physiological conditions [6]. To date, the interrelation between substrate load and transport activity is largely unknown. Therefore, the aim of this study was to investigate the role of primary bile salts in short-term feedback regulation of sinusoidal substrate uptake in perfused rat liver.

## Materials and methods

Transporter retrieval from the plasma membrane was analyzed by assessing subcellular distribution of sinusoidal and canalicular transporters in immunofluorescence images from tissue sections of perfused rat liver by an automated image analysis method, as described previously [6,7]. Shortly, livers were perfused with 100 µmol/L of taurocholate (TC), taurochenodeoxycholate (TCDC) or control buffer for 60 min. Sample preparation and immunostaining were performed according to standard operating procedures. In confocal images, basolateral or canalicular membranes, respectively, were identified by foreground-background detection and fluorescence intensity profiles were extracted perpendicular to short membrane segments. To determine changes in subcellular transporter distribution, intensity profiles from liver samples before and after perfusion were compared statistically. In addition, co-localization of endosome markers and basolateral bile salt transporters was analyzed in confocal images by calculation of the weighted co-localization coefficient from co-localizing pixels according to a preset threshold. In functional studies, net substrate uptake was monitored in perfused rat liver by a pulse chase technique. Rat livers were continuously perfused with 100 µmol/L of TC. Additionally, livers were pre-perfused with 100 µmol/L of TCDC or TC, followed by a wash out-phase and a bolus of ^3^[H]-TC. Substrate uptake was monitored by detection of radioactivity in effluent and bile. The volume of bile flow was measured. In addition, the involvement of signaling molecules was analyzed in inhibitor assays. Inhibitors were added 20 min before bile salt stimulation throughout the experiment [6].

## Results

As shown by automated image processing, perfusion of rat liver with TCDC, but not with TC or control buffer, induced a significant change in subcellular localization of Ntcp. Furthermore, co-localization studies revealed an increase of Ntcp in EEA1 (early endosome antigen 1)-positive early endosomes. However, the subcellular distribution of the canalicular bile salt export pump Bsep remained unchanged, which indicates that the canalicular bile salt secretion is unaffected by bile salt perfusion [6]. Based on these data, the localization of the Oatp transporters was analyzed. Interestingly, similar to Ntcp significant Oatp1a1 internalization was demonstrated by automated image evaluation. However, in contrast to Ntcp, Oatp1a1 internalization was detectable irrespective of the perfused bile salt, but was absent under control conditions. This indicates differential regulatory mechanisms for Ntcp and Oatp1a1 [8]. While under control conditions, Ntcp and Oatp1a1 show homogenous expression between periportal and perivenous hepatocytes, Oatp1a4 and Oatp1b2 show a gradient zonation with predominant expression in perivenous hepatocytes. Therefore, for these proteins, not only subcellular, but also zonal distribution was analyzed by automated image processing. However, both, subcellular as well as zonal distribution of Oatp1a4 and Oatp1b2 remained unchanged under all three conditions [8].

These results correlated well with functional studies. TCDC, but not TC, significantly increased the amount of ^3^[H]-TC in the effluent, indicating a TCDC-dependent reduction in sinusoidal net TC-uptake. Together with the data from automated image analysis this indicates a TCDC-induced reduction of V_max_ by internalization of Ntcp from the basolateral membrane. As shown by inhibitor assays, this effect is mediated by protein kinase C and protein phosphatase 2B. Phosphoinositide 3-kinase was identified as a constitutively active mediator in the regulation of sinusoidal bile salt uptake [6].

To confirm the role of primary bile salts in the short-term regulation of Oatp1a1 on a functional level, estone-3-sulfate (E-3-S) was selected as a substrate, as it is predominantly transported by proteins from the Oatp family. Experimental settings for pulse-chase experiments with ^3^[H]-E-3-S were defined according to the protocol described above. In line with the localization data, these functional studies showed a similar reduction of net ^3^[H]-E-3-S-uptake by primary bile salts (TC or TCDC, respectively), as compared to control conditions. Furthermore, results from inhibitor assays indicate differential regulation of Ntcp and Oatp1a1 by primary bile salts. While protein kinase C was identified as a mediator in the regulation of sinusoidal TC-uptake by TCDC, its inhibition had no effect on the regulation of sinusoidal E-3-S uptake by TC or TCDC, respectively.

## Conclusions

Taken together, we propose a role of primary bile salts in the feedback regulation of sinusoidal substrate uptake. The interrelation between substrate load and transporter activity indicates a new physiological level of regulation. Under an increased portal bile salt load rapid internalization of transporter proteins into vesicles may adjust substrate uptake along the acinus.
